# Characterization of an Additional Splice Acceptor Site Introduced into *CYP4B1* in *Hominoidae* during Evolution

**DOI:** 10.1371/journal.pone.0137110

**Published:** 2015-09-10

**Authors:** Eva M. Schmidt, Constanze Wiek, Oliver T. Parkinson, Katharina Roellecke, Marcel Freund, Michael Gombert, Nadine Lottmann, Charles A. Steward, Christof M. Kramm, Vladimir Yarov-Yarovoy, Allan E. Rettie, Helmut Hanenberg

**Affiliations:** 1 Department of Pediatric Hematology, Oncology and Clinical Immunology, Children’s Hospital, Heinrich Heine University, 40225 Düsseldorf, Germany; 2 Department of Otorhinolaryngology and Head/Neck Surgery, Heinrich Heine University, 40225 Düsseldorf, Germany; 3 Department of Medicinal Chemistry, School of Pharmacy, University of Washington, Seattle, WA 98195, United States of America; 4 Wellcome Trust Sanger Institute, Cambridge, United Kingdom; 5 Division of Pediatric Hematology and Oncology, Department of Child and Adolescent Health, University of Göttingen, 37099 Göttingen, Germany; 6 Departments of Physiology and Membrane Biology, University of California Davis, Davis, CA 95616, United States of America; 7 Department of Pediatrics, Indiana University School of Medicine, Indianapolis, IN 46202, United States of America; 8 Department of Pediatrics III, University Children’s Hospital Essen, University of Duisburg-Essen, 45122 Essen, Germany; Institute of Zoology, Chinese Academy of Sciences, CHINA

## Abstract

CYP4B1 belongs to the cytochrome P450 family 4, one of the oldest P450 families whose members have been highly conserved throughout evolution. The CYP4 monooxygenases typically oxidize fatty acids to both inactive and active lipid mediators, although the endogenous ligand(s) is largely unknown. During evolution, at the transition of great apes to humanoids, the CYP4B1 protein acquired a serine instead of a proline at the canonical position 427 in the meander region. Although this alteration impairs P450 function related to the processing of naturally occurring lung toxins, a study in transgenic mice suggested that an additional serine insertion at position 207 in human CYP4B1 can rescue the enzyme stability and activity. Here, we report that the genomic insertion of a CAG triplet at the intron 5–exon 6 boundary in human *CYP4B1* introduced an additional splice acceptor site in frame. During evolution, this change occurred presumably at the stage of *Hominoidae* and leads to two major isoforms of the CYP4B1 enzymes of humans and great apes, either with or without a serine 207 insertion (insSer207). We further demonstrated that the CYP4B1 enzyme with insSer207 is the dominant isoform (76%) in humans. Importantly, this amino acid insertion did not affect the 4-ipomeanol metabolizing activities or stabilities of the native rabbit or human CYP4B1 enzymes, when introduced as transgenes in human primary cells and cell lines. In our 3D modeling, this functional neutrality of insSer207 is compatible with its predicted location on the exterior surface of CYP4B1 in a flexible side chain. Therefore, the Ser207 insertion does not rescue the P450 functional activity of human CYP4B1 that has been lost during evolution.

## Introduction

Cytochrome P450s (CYPs) constitute a large superfamily of genes that have co-evolved with their hosts—higher plants, prokaryotic and eukaryotic organisms—from a single common ancestor [1, 2]. In humans, the HUGO Gene Nomenclature Committee currently lists 107 genes and pseudogenes (http://www.genenames.org/genefamilies/CYP) belonging to 4 major and 13 smaller P450 families [3]. Although there are remarkably similar substrate specificities for the vast majority of P450 orthologs in mammals due to semi-conserved active-site sequences and ligand access channels [4], human *CYP4B1* stands out as a functionally unusual member of this single-gene subfamily (reviewed in [5]).

CYP4B1 was initially recognized as the P450 enzyme responsible for the activation of a naturally occurring pro-toxin, 4-ipomeanol (4-IPO), to an alkylating agent [6–10]. 4-IPO is produced by sweet potatoes (*Ipomoea batatas*) infected with the fungus *Fusarium solani* [11–14]. When livestock ingest moldy sweet potatoes, 4-IPO uptake induces selective cellular toxicity in the lungs as the primary organ affected [11, 12, 14] where 70% of all CYP4B1 transcripts are expressed [15–18]. Based on the preclinical observations in rodents and dogs that doses can be established where the cytotoxicity is restricted to the lungs and the findings that human lung cancer cells *in vitro* and *in vivo* can be specifically targeted (reviewed in [19]), three human clinical phase I/II trials were conducted [20–22]. Surprisingly, dose-escalation studies revealed that no lung toxicity, but some reversible dose-dependent liver toxicity occurred after intravenous administration of 4-IPO. However, no objective anti-tumor effects were noted, for either lung or liver cancer patients [20–22].

Major interspecies differences in the activity of CYP4B1 enzyme between the human and other mammalian homologs have been reported (reviewed in [5]). When overexpressed in human HepG2 liver cells, the native human CYP4B1 enzyme is unable to metabolize the classical substrates such as 2-aminoanthracene (2-AA) [23, 24] and 4-IPO [9, 25]. In addition, no catalytic activity of the human enzyme was detected upon its expression in insect cells using a baculovirus system [26], presumably because the human enzyme sequence carries serine instead of proline at position 427 in the meander region near the heme-binding site [26, 27]. Remarkably, the presence of serine instead of proline at position 427 is specific to human CYP4B1 and also unique among other human CYP450 enzymes [5, 28].

In addition to the initially identified human cDNA sequence [25], three groups of investigators reported an in-frame insertion in the major *CYP4B1* transcript isolated from several human tissues [29–31]. Bylund *et al*. performed RT-PCR studies on four human seminal vesicle samples and detected a mixture of the native CYP4B1 and an isoform with a 3-bp insertion (CAG) encoding serine at position 207 in all samples [29]. Carr *et al*. suggested that the serine insertion is the result of an alternatively splice transcript but was not able to find a transcript without serine at position 207 in five bacterial clones amplified from human lung [31]. In these studies, the functional significance of the serine insertion was not determined, because it was not possible to express active human CYP4B1 enzyme, either with or without the serine insertion [31]. In 2001, Imaoka *et al*. reported that the serine insertion was carried by the *CYP4B1* ‘gene’ in 50 different individuals [30], suggesting that it is the major transcript in humans, although no supporting data were presented. This group was also able to express human CYP4B1 insSer207 in the liver of a transgenic mouse and as a fusion protein of CYP4B1 and NADPH P450 reductase in yeast cells [30]. The microsomal preparations containing the human protein catalyzed ω-hydroxylation of lauric acid and activated 2-AF, both known substrates for CYP4B1, thus prompting the authors to hypothesize that the serine insertion “may also stabilize the native CYP4B1” [30]. However, these findings regarding a functional activity of native human CYP4B1 with the serine insertion towards classical substrates of animals orthologs have not been confirmed by others (reviewed in [5]) and are in sharp contrast to the results of phase I/II dose escalation trials in humans in which 4-IPO was administered as an anti-lung cancer agent [20–22]. Although humans express 70% of all *CYP4B1* transcripts in the lung [29–32], no lung toxicity was observed with administration of 4-IPO doses that are highly toxic/lethal in animals [17, 19, 33, 34].

To clarify some of these issues, we recently re-engineered human CYP4B1 for efficient activation of 4-IPO by introducing amino acid residues from the highly active rabbit enzyme into the native human protein as well as the p.S427P CYP4B1 mutant [28]. Using lentiviral vector systems to achieve stable expression of human and rabbit CYP4B1 in human HepG2 liver cells and primary T cells, we demonstrated that native human CYP4B1 is an inactive and relatively unstable protein with a short half-life compared to its rabbit homolog. Exchange of serine with proline at position 427 improved the half-life of the human CYP4B1 protein and dramatically increased its enzymatic activity towards 4-IPO, although this single point mutant was still less active than the rabbit enzyme. Eventually, through systematic mutagenesis, we identified 12 additional amino acid substitutions that conferred all of the functional activity of rabbit CYP4B1 to human CYP4B1 [28]. These prior efforts establish a baseline against which to probe the functional consequences of insSer207 in CYP4B1.

In the present study, we analyzed the molecular basis for serine insertion at position 207 in the human CYP4B1 enzyme and determined the frequency of insSer207 in human *CYP4B1* transcripts. In order to determine whether insSer207 could have emerged as a compensatory mechanism for reduced P450 enzymatic activity due to a p.P427S exchange that had occurred in humans, we tested whether insSer207 stabilizes the half-life and/or enhances the functional activity of native human CYP4B1 and the p.S427P CYP4B1 mutant proteins. Finally, we explored the evolution of the human *CYP4B1* genomic sequence through comparison of insSer207 and p.P427S in humans to those in great apes and non-human primates as well as other mammals.

## Materials and Methods

### Serine insertion in normal human *CYP4B1* mRNA

**Fig 1:**
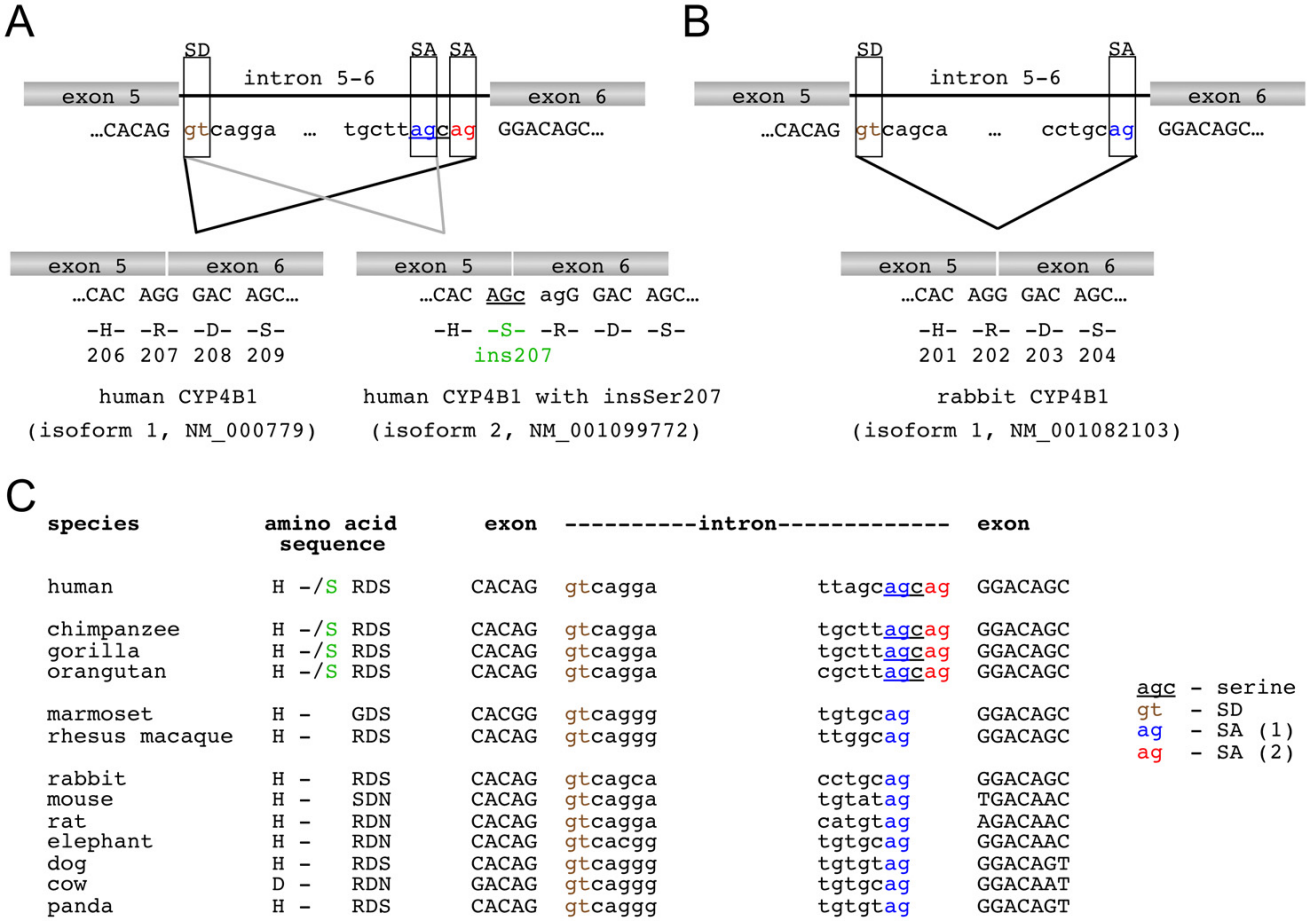
Alternative Splicing of *CYP4B1*. (A) Human *CYP4B1*. The human *CYP4B1* intron 5–6 harbors two splice acceptors (SAs) in frame allowing the generation of two alternative *CYP4B1* transcripts, with (isoform 2: NM_001099772) or without (isoform 1: NM_000779) insSer207. (B) Rabbit *CYP4B1*. Intron 5–6 of rabbit *CYP4B1* carries only a single SA and therefore generates a single transcript without insSer202 (isoform 1: NM_001082103). (C) Alignment of *CYP4B1* sequences from other species. Alignment of the genomic DNA of *CYP4B1* from other species shows that the insertion of the CAG triplet, which generates the additional splice acceptor site, exists only in humans and great apes, and not in rhesus, macaque, marmoset or other mammals.

**Fig 2:**
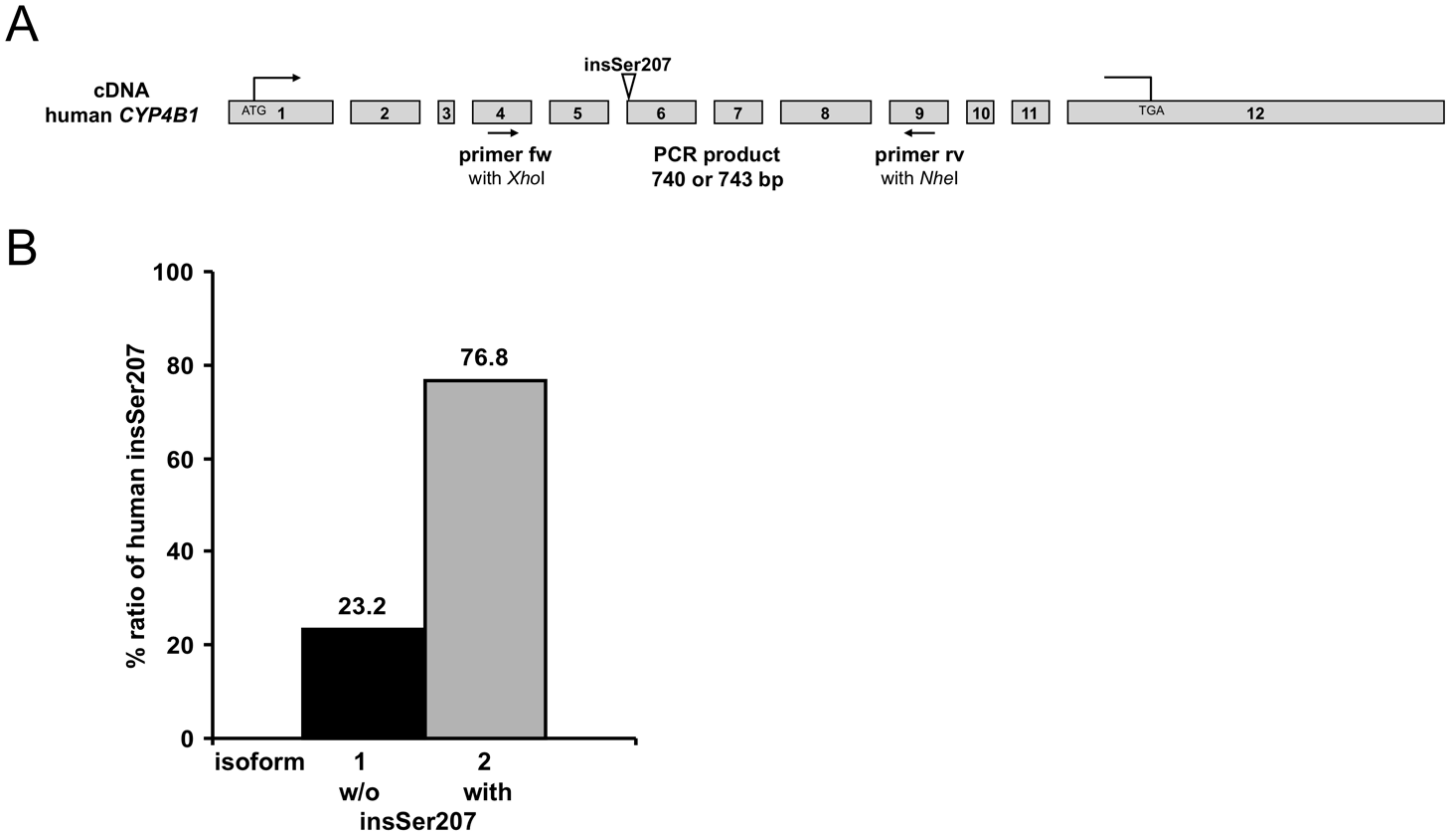
Frequency of insSer207 in human *CYP4B1* transcripts. (A) Schematic outline of *CYP4B1*. The forward (fw) primer binds in exon 4 and the reverse (rv) primer in exon 9, thus resulting in PCR products of 240 bp and 243 bp, respectively. (B) Frequency of human CYP4B1 without (w/o) and with insSer207. The plasmid DNA from 220 bacterial colonies was isolated and sequenced. The percentages of CYP4B1 transcripts with and without (w/o) insSer207 are shown.

**Fig 3:**
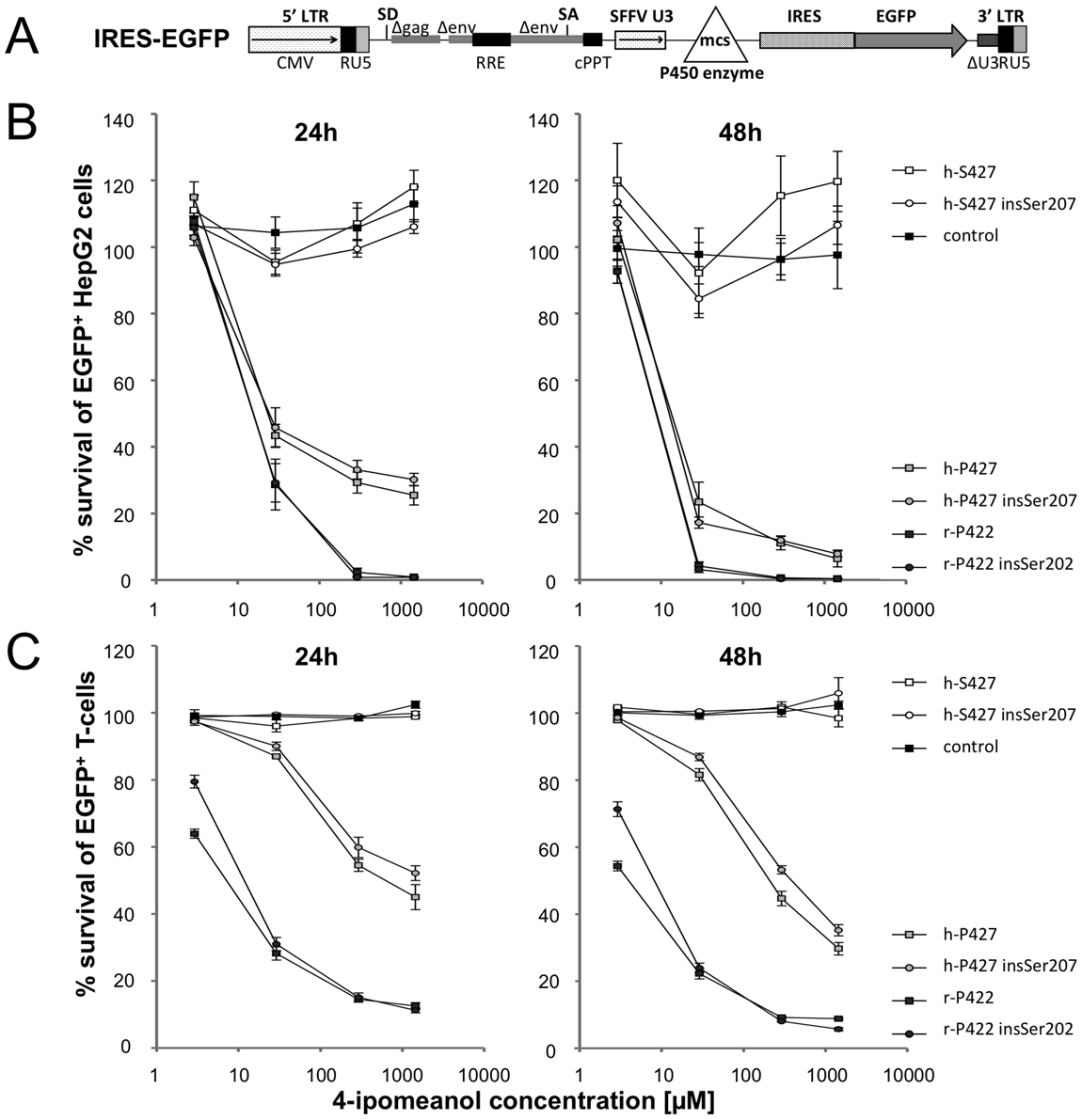
Functional analysis of CYP4B1. (A) Schematic outline of the lentiviral (LV) vectors used for expression of CYP4B1. For functional testing, the different CYP4B1 forms were cloned into a LV vector with an IRES-EGFP site. CMV: CMV promoter; SD: splice donor; LTR: long terminal repeat; SA: splice acceptor; RRE: Rev responsive element, cPPT: central polypurine binding tract; SFFV U3: U3 promoter of the spleen focus-forming virus; mcs: multicloning site; IRES: internal ribosomal entry site; EGFP: enhanced green fluorescent protein. (B) Toxicity of HepG2 cells after 4-IPO exposure. Survival of EGFP+ HepG2 cells (%) after 24 and 48 h of incubation with 0, 2.9, 29, 290 or 1450 μM 4-IPO as measured by flow cytometry. The cells stably expressed the different CYP4B1 enzymes: h-S427: human wild-type (isoform 1); h-S427 insSer207: human wild-type with insSer207 (isoform 2), h-P427: human mutated p.S427P (artificial); h-P427 insSer207: human mutated p.S427P with insSer207 (artificial); r-P422: rabbit wild-type (isoform 1); r-P422 insSer202: rabbit with insSer202 (artificial). For each construct, the mean ± standard error of the mean (SEM) values are shown from at least three independent experiments. The transduction efficiency of HepG2 cells (not shown) was ≥90%, as determined by EGFP expression in flow cytometry. (C) Toxicity of T cells after 4-IPO exposure. Survival of EGFP+ primary human T cells (%) expressing the different CYP4B1 proteins [listed for (B)] after 24 and 48 h of incubation with increasing doses of 4-IPO as assessed by flow cytometry. For each construct, the mean ± SEM values are shown from at least three experiments.

To assess the frequency of the serine insertion in human *CYP4B1* mRNA (Fig 1A), a PCR was performed on human reference cDNA (Clontech #636690, Lot #1005341A) using the exon 4 forward primer 5'-GC**CTCGAG**GGTTGCAGCACCGCAAGCTG with the *Xho*I restriction enzyme recognition site (bold) and the exon 9 reverse primer 5'- GG**GCTAGC**CACAGGTGGGTAGAGGCGG with the *Nhe*I site (Fig 2A). PCR products of 740 and 743 bp were ligated into the multiple cloning site (mcs) of the lentiviral vector IRES-EGFP with an internal ribosome entry site—enhanced green fluorescent protein (Fig 3A) and then transformed into Top10 bacteria (Invitrogen, Karlsruhe, Germany). Plasmid DNA was isolated from 220 bacterial clones and sequenced using the forward primer 5'- GGACCTGAAATGACCCTGCG that binds in the spleen focus-forming virus (SFFV) promoter.

### Cell cultures and primary cells

Human embryonic kidney (HEK293T) cells and human hepatoma (HepG2) cells were purchased from ATCC (Manassas,VA), and human fibrosarcoma HT1080 cells were obtained from DSMZ (Braunschweig, Germany). All cells were grown in Dulbecco’s Modified Eagle’s Medium supplemented with penicillin (100 U/mL), streptomycin (100 μg/mL), 2 mM glutamine (all from Gibco/BRL, Karlsruhe, Germany), and 10% heat-inactivated fetal bovine serum (PAN, Aidenbach, Germany) at 37°C in a humidified atmosphere with 5% CO_2_.

Primary human T lymphocytes from peripheral blood (PB) samples from healthy adult volunteers who provided written informed consent that is documented in the department of Otorhinolaryngology & head/neck surgery (ENT). This study including the documentation was approved by the ethics committee of the Heinrich-Heine-University of Düsseldorf (ethics No. 4687). After Ficoll-Hypaque density gradient centrifugation, outgrowth of >95% T cells was achieved by incubation of the mononuclear cells on immobilized CD3 and CD28 antibodies (BD Bioscience, San Jose, CA) in combination with IL-2 (100 IU/ml, Chiron, Marburg, Germany) in complete Iscove’s Modified Dulbecco’s Medium (Sigma-Aldrich, Deisenhofen, Germany) as described previously [28, 35, 36].

### Plasmid construction, lentivirus production, and cell transduction

**Fig 4:**
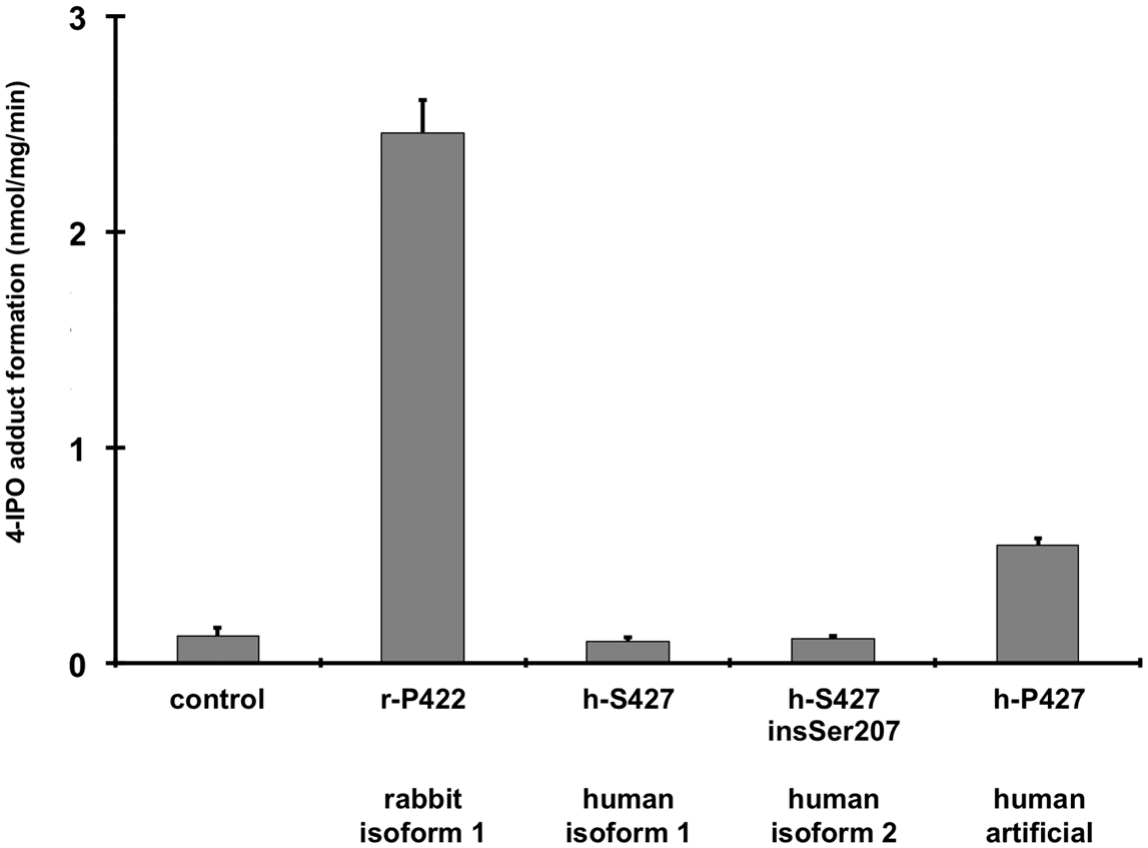
Bioactivation of 4-IPO to a stable NAC/NAL adduct. The NADPH-dependent formation of NAC/NAL adducts by membranes isolated from HepG2 cells stably transduced with the different CYP4B1 cDNAs r-P422: rabbit wild-type (isoform 1); h-S427: human wild-type (isoform 1); h-P427: human mutated p.S427P (artificial); h-S427 insSer207: human wild-type with insSer207 (isoform 2) after incubation in 50 nM 4-IPO for 20 minutes is shown. The adduct formation rate is shown as the mean ± standard deviation (SD) of triplicate incubations.

**Fig 5:**
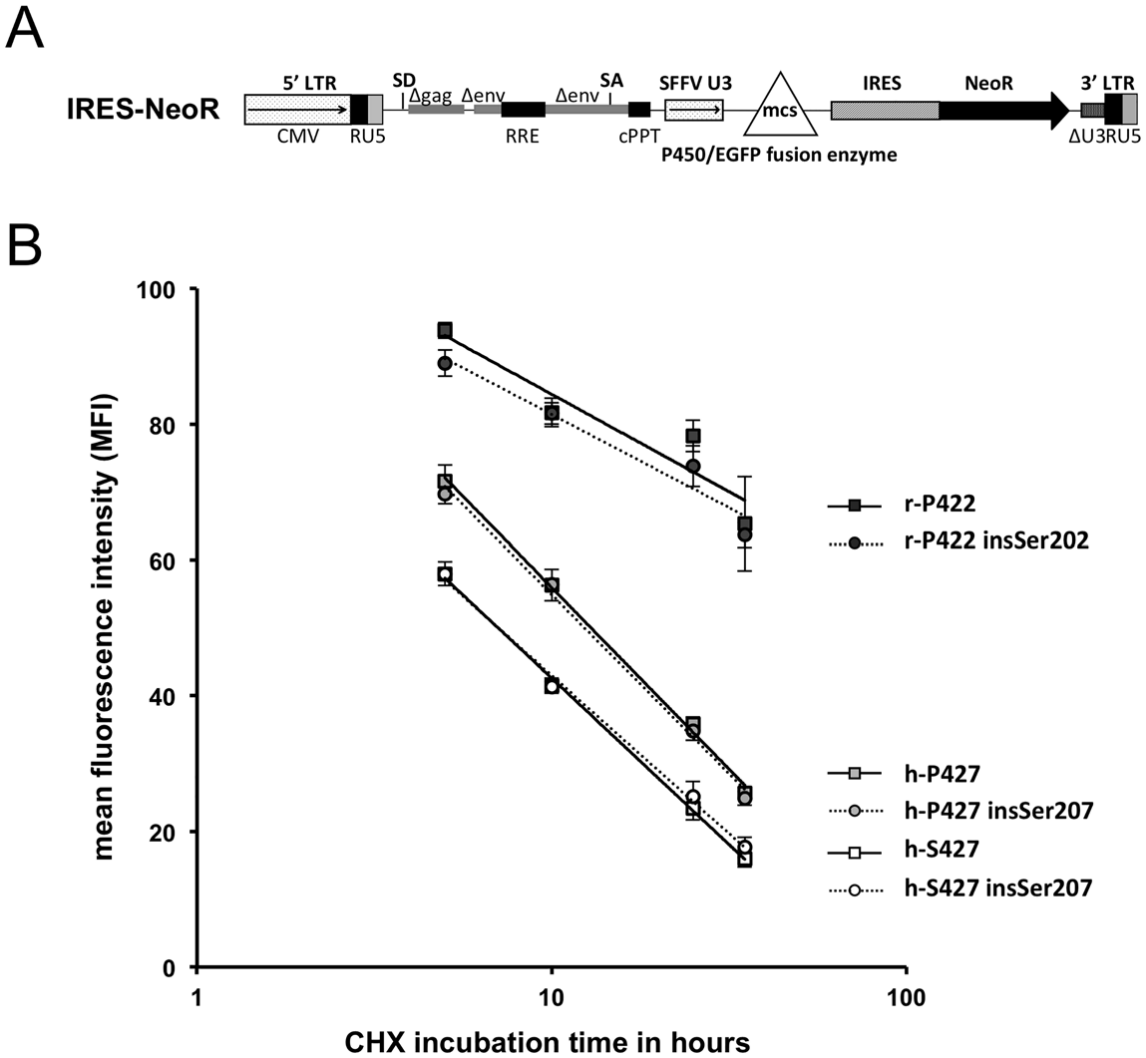
Protein stability of CYP4B1. (A) Schematic outline of the lentiviral vector used for expression of CYP4B1. CMV: CMV promoter; SD: splice donor; LTR: long terminal repeat; SA: splice acceptor; RRE: Rev responsive element, cPPT: central polypurine binding tract; SFFV U3: U3 promoter of the spleen focus-forming virus; mcs: multicloning site; IRES: internal ribosomal entry site; neoR: neomycin phosphotransferase (nptII) resistance cDNA; EGFP: enhanced green fluorescent protein. (B) Protein half-life analysis of CYP4B1 isoforms in HepG2 cells. Protein half-life analysis was performed by adding 50 μg/ml cycloheximide (CHX) to the transduced and G418-selected HepG2 cells. The mean fluorescence intensity (MFI) of CYP4B1/EGFP fusion proteins was assessed at various time points by flow cytometry. For each construct, the mean ± SEM values are shown from at least three experiments.

The two lentiviral expression plasmids IRES-EGFP (used in Fig 3A and for Fig 4) and IRES-NeoR (Fig 5A) as well as expression constructs for the native rabbit CYP4B1 (NM_001082103, isoform 1), the wild-type human CYP4B1 (NM_000779, isoform 1), and the mutant human p.S427P were described previously [28]. For insertion of serine at position 207 in the two human CYP4B1 proteins (NM_001099772: wild-type with insertion, isoform 2) and at position 202 in the rabbit enzyme, a commercial site-directed mutagenesis kit (Stratagene, La Jolla, CA) was used according to the manufacturer’s recommendations with the following primers 5'-CGGCCTGGGCCACAGCAGGGACAGCAGCTACTACC (forward) and 5'-GGTAGTAGCTGCTGTCCCTGCTGTGGCCCAGGCCG (reverse) for human *CYP4B1* and 5'-GGAGACAGTGGCCTGAATCACAGCAGGGACAGCAGCTACTACGTG (forward) and 5'- CACGTAGTAGCTGCTGTCCCTGCTGTGATTCAGGCCACTGTCTCC (reverse) for rabbit *CYP4B1*. The cDNAs with a serine insertion, either with or without a 3'EGFP fusion, were then cloned into the IRES-NeoR and IRES-EGFP constructs, respectively, using the *Nhe*I-*Bam*HI restriction enzyme sites in the vectors. The final lentiviral expression constructs were verified by Sanger sequencing.

Vesicular stomatitis virus G (VSV-G) pseudotyped replication-deficient lentiviral particles were then produced after transfection into HEK293T cells and cryopreserved as described previously [28, 37]. Functional titers of the supernatants were assessed on HT1080 cells. HepG2 cells were transduced with either IRES-EGFP or IRES-NeoR vectors using 8 μg/ml polybrene (Sigma-Aldrich) [28, 37]. Primary human T cells were transduced in the presence of 100 U/ml IL-2 on the recombinant fibronectin fragment CH-296 (Takara Shuzo, Otsu, Japan) as previously described [28, 35, 38, 39].

### Toxicity assay with 4-IPO

Five days after transduction of the HepG2 cells and four days after transduction of the T lymphocytes, cells were incubated in complete medium with increasing concentrations of 4-IPO (0, 2.9, 29, 290, and 1450 μM). Cultures were harvested after 24 and 48 h, centrifuged, and then re-suspended in PBS with propidium iodide (PI; Sigma-Aldrich). Each sample was then analyzed by flow cytometry on a FACSCalibur (BD Biosciences, Heidelberg, Germany), and 10,000 events were recorded. Live/dead cell analysis of EGFP+ cells was then performed using the CellQuest software as previously described [28]. Each experiment was performed independently at least three times.

### CYP4B1-dependent NAC/NAL adduct formation and quantitative analysis

*In vitro* bioactivation of 4-IPO by human CYP4B1 enzyme was assessed by incubating membrane preparations (1 mg) from transduced stably expressing HepG2 cells in the presence of substrate (1 mM), NADPH (1 mM), and both N-acetylcysteine (NAC) and N-acetyllysine (NAL) at 5 mM concentrations. Incubations were conducted at 37°C for 30 min, and adduct formation was quantified by a high performance liquid chromatography ultraviolet detector as previously described [40]

### Protein half-life analysis

For determination of the protein half-lives, we used 3'EGFP-fusion CYP4B1 proteins in the lentiviral IRES-NeoR vector (Fig 5A) as described previously [28]. Briefly, G418-resistant HepG2 cells were incubated with 50 μg/ml cycloheximide (CHX, Sigma-Aldrich), which inhibits eukaryotic protein translation. After 5, 10, 25, and 35 h, cells were harvested and fixed in 4% paraformaldehyde and, the mean fluorescence intensity (MFI) was assessed by flow cytometry. The MFI values of cells treated with CHX were expressed as a percentage of the MFI of cells incubated in medium without CHX. Each experiment was performed independently at least three times.

### Modeling of human CYP4B1

Structural modeling of human CYP4B1 with insSer207 was performed using the Robetta server [41–43] that used the X-ray structure of a bacterial mimic of CYP2C9 (pdb id: 3QI8) as a template. Sequence identity between human CYP4B1 and the bacterial mimic of CYP2C9 was ~24%. A total of 5,000 models were generated followed by model clustering. The best CYP4B1 models were chosen based on the centers of the largest clusters and defined by having the lowest standard mean deviation value (between corresponding positions of C-α atoms of all residues) among all other models in a cluster.

## Results

### Evolution of the additional splice acceptor site in *CYP4B1*

To understand the molecular basis for alternative *CYP4B1* transcripts that do or do not encode insSer207, we first analyzed the splice donor and acceptor sites in intron 5 of human and rabbit *CYP4B1*. As shown in Fig 1A, the human genome harbors two splice acceptor sites in frame before exon 6, thus allowing the generation of two alternative transcripts, with or without serine at position 207, respectively. In contrast, intron 5 of the rabbit gene (Fig 1B) carries only a single splice acceptor site at the corresponding exon 6 boundary and therefore generates a single transcript without serine at amino acid position 202. Next, we aligned the genomic DNA of the corresponding *CYP4B1* intron 5 sequences of great apes, monkeys, and other mammals obtained from the ensembl genome database (www.ensembl.org). The alignment in Fig 1C revealed that an insertion of a CAG triplet, generating the additional splice acceptor site, occurred during evolution at the level of *Hominoidae* (*Homo sapiens* and *apes*) and clearly distinguishes these species from old world (*rhesus macaque*) and new world (*marmoset*) monkeys. Other mammals such as rat, cow, and elephant also do not have the alternative splice acceptor site, thus making insSer207 specific for *Hominoidae*.

### Ratio of *CYP4B1* transcripts in healthy human tissues

To determine the usage of the two different splice acceptor sites of *CYP4B1* in human transcripts, a PCR with the primer pair shown in Fig 2A was performed on a commercial human cDNA library that was pooled from the mRNA of various organs obtained from 81 healthy donors. The PCR products were cloned into a high copy number plasmid, and the DNA inserts in bacterial colonies were isolated and sequenced. As shown in Fig 2B, 169 out of 220 (76.8%) bacterial clones contained insSer207, whereas 51 clones (23.2%) did not. Thus, the majority of translated human CYP4B1 contains insSer207.

### Functional analysis of human and rabbit CYP4B1 with/without insSer207/202

The enzymatic activation of 4-IPO is an established tool for assessing and comparing the activity of CYP4B1 orthologs. To evaluate the functional consequences of insSer207/202 on the activity of the CYP4B1 enzyme, PCR was used to introduce the CAG sequence, containing the additional splice acceptor site, into the human native and mutant p.S427P CYP4B1 enzymes at the intron 5–exon 6 boundary. We also introduced the serine insertion at the corresponding position 202 of the native rabbit CYP4B1 protein (insSer202). For stable expression in mammalian cells, all six cDNAs were cloned into the lentiviral expression vector IRES-EGFP (Fig 3A), thus linking the expression of the *CYP4B1* cDNAs to EGFP as a marker gene for transduced cells [28]. Replication-deficient lentiviral particles pseudotyped with VSV-G were titered as described previously [28, 37] and used to transduce human HepG2 liver cells with the different constructs at similar viral particle per target cell, also called multiplicities-of-infection (MOIs) of approximately 5–7. The results demonstrated that HepG2 cells transduced with the control vector did not show any cell death in response to the increasing 4-IPO concentration after 24 and 48 h (Fig 3B), because bioactivation could not occur. Moreover, as shown previously [28], the native human CYP4B1 enzyme was not capable of processing 4-IPO, while both the rabbit wild-type and the human p.S427P mutant CYP4B1 induced clear cytotoxic effects on the cells. Finally, introduction of serine at corresponding positions into the wild-type rabbit or human p.S427P CYP4B1 did not alter the high activities of both enzymes towards 4-IPO (Fig 3B).

To confirm these findings in non-transformed cells that do not express *CYP4B1* mRNA (Wiek & Hanenberg, unpublished), primary human T cells from healthy donors were isolated, transduced with the same vectors at similar MOIs, and then challenged with increasing doses of 4-IPO. After 24 and 48 h, the cells were harvested, and the survival of transduced EGFP+ T cells was analyzed by flow cytometry. As shown in Fig 3C, the insSer207/202 did not restore the negligible activity of the native human CYP4B1 enzyme nor alter the strong metabolizing activities of the wild-type rabbit or the mutant human p.S427P CYP4B1 proteins. Therefore identical to the results obtained in HepG2 cells, the serine insertion did not alter the enzymatic activity of neither the wild-type rabbit and human nor the mutant human CYP4B1 protein.

### 4-IPO induced adduct formation and cellular cytotoxicity

As survival of stably transduced human cells was used as the indicator for CYP4B1 enzyme activity towards 4-IPO, we wanted to confirm that a direct correlation exists between the percentage of EGFP+ cells that survived after 4-IPO incubation and 4-IPO adduct formation in these cells. To this end, we assessed NAC/NAL adduct formation in membrane preparations from HepG2 cells stably transduced with the rabbit or one of the three human *CYP4B1* cDNAs. Just as 4-IPO was not toxic to cells expressing wild-type human CYP4B1 (Fig 3B), membranes prepared from these cells did not catalyze the bioactivation of 4-IPO (Fig 4). However, when the serine in the meander region was reverted to the evolutionarily conserved proline at position 427 (p.Ser427Pro), 4-IPO was bioactivated and formed NAC/NL adducts (Fig 4). The very active rabbit enzyme served as a positive control. The results clearly demonstrated that both assays, cell survival and NAC/NAL adduct formation, are well suited for measuring the 4-IPO metabolizing activity of different CYP4B1 enzymes. Importantly, both assays also showed that the insSer207 variant of human CYP4B1 does not ‘rescue’ the loss of enzyme activity towards xenobiotics caused in large part by the presence of the meander region amino acid, Ser427.

### Protein stability of human and rabbit CYP4B1 with or without serine insertion

We have previously shown that the differences in the 4-IPO metabolizing activity of the wild-type rabbit and human mutant CYP4B1 enzymes is caused, at least in part, by differences in protein half-life and expression level [28]. Given that Imaoka *et al*. suggested that the function of insSer207 may be to stabilize the native human CYP4B1 enzyme [30], we next tested the effect of this insertion on the half-lives and expression levels of the native rabbit and human CYP4B1 proteins. We also included the human CYP4B1 p.S427 mutant with and without insSer207 as additional controls.

As no antibody is available that can recognize both the human and the rabbit proteins equally well, 3′EGFP-CYP4B1 fusion proteins were cloned into a lentiviral vector with an IRES-NeoR cassette (Fig 5A) as described previously [28]. HepG2 cells were transduced at similar MOIs of approximately 3 with the infectious lentiviral particles stably expressing the different fusion enzymes and then selected with G418 (Geneticin^®^). G418-resistant HepG2 cells were incubated with cycloheximide (CHX) and then harvested at several time points to semi-quantitatively assess the mean fluorescent intensities (MFIs) of the CYP4B1-EGFP fusion constructs by flow cytometry. Similarly to our previously reported findings [28], HepG2 cells expressing the native rabbit CYP4B1 showed the highest MFI (500 on the FL1 channel) of all EGFP-tagged proteins and the smallest decline during the observation period (t_1/2_ >75 h). These characteristics of the wild-type rabbit protein were not influenced by insSer202. The analysis further demonstrated that the corresponding insSer207 in the human protein also did not influence the half-life and expression level of either the native (t_1/2_ ~7h) or the mutant p.S427P (t_1/2_ ~13h) CYP4B1 protein (Fig 5B, pairs of solid versus hatched lines).

### Structural models of the serine insertions in human and rabbit CYP4B1

**Fig 6:**
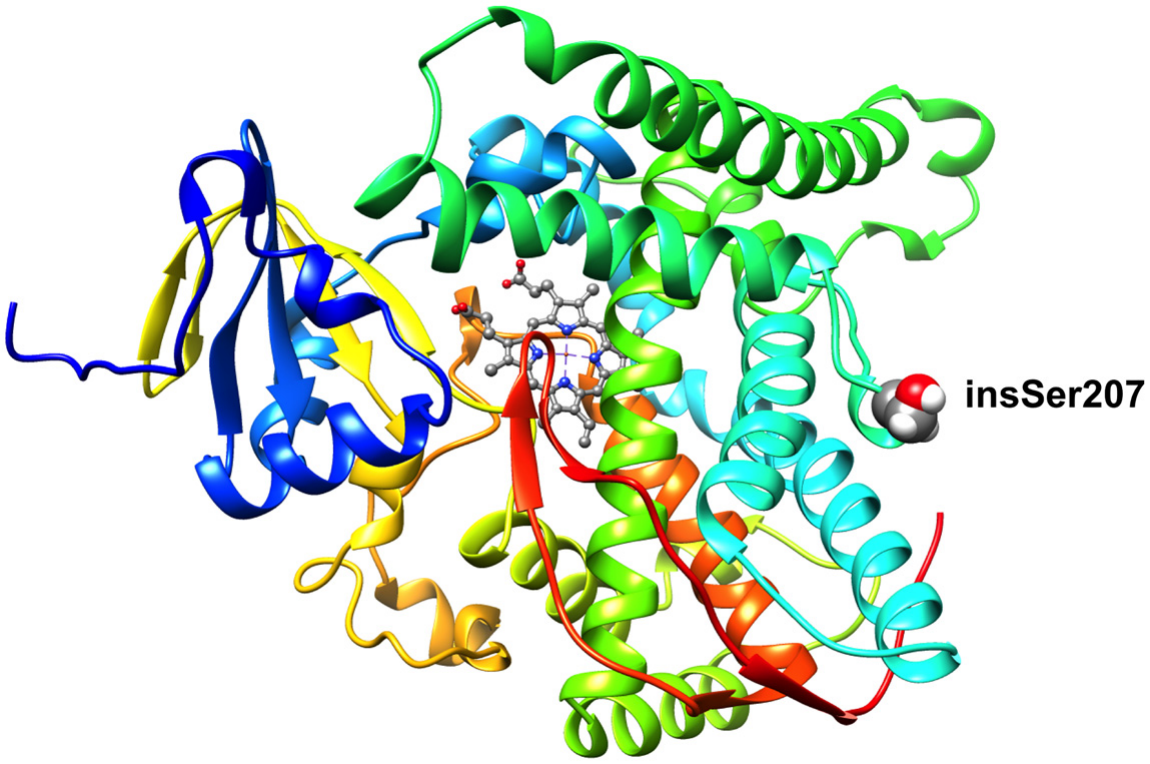
Structural model of human CYP4B1 with insSer207 located in a loop region from G198 to S210. Cartoon representation of the structure of human CYP4B1. The side chain of insSer207 and the heme prosthetic group are shown as spacefilling and ball and stick representations, respectively. Carbon atoms are colored gray, oxygen atoms are colored red, nitrogen atoms are colored blue, and hydrogen atoms are colored white. The figure was generated using UCSF Chimera [43].

Finally, to better understand why insSer207 had no effect on the expression level, half-life, or enzymatic activity of the three CYP4B1 proteins, we generated a three-dimensional model for insSer207 in human CYP4B1. Because no crystal structure is available for any CYP4 protein, we used the Rosetta-based Robetta server to generate a homology model. The model shown in Fig 6 localizes insSer207 to a loop region, distant from the heme catalytic center, that is comprised by residues G198 to S210. Within this loop, the Ser207 side-chain is oriented away from the active site towards the soluble environment. These structural features are consistent with insSer207 having a minimal effect on human CYP4B1 structure and catalysis. Similar structural considerations also apply for the rabbit wild-type and human p.S427P CYP4B1 proteins (not shown).

## Discussion

Human CYP4B1 has been described as an enigmatic enzyme [5], in part due to the two human-specific serine residues that have been incorporated into the primary sequence during evolution. One unanswered question regarding CYP4B1 in the literature is the importance of insSer207 in human CYP4B1 and its role in the function of the enzyme. Although not described in the initial cloning of the human *CYP4B1* cDNA [25], three groups subsequently described insSer207 as the major form of *CYP4B1* mRNA in human tissues [29–31]. Carr *et al*. reported that only this insertion was present in 5 out of 5 bacterial clones, but a more in-depth semi-quantification of the ratio of the two isoforms was not performed in any of the earlier studies. In the present work, after sequencing a library of exon 5-exon 6 amplified *CYP4B1* cDNA fragments, we demonstrated that the insSer207 is encoded by 76% of *CYP4B1* transcripts in humans. This insertion is the consequence of a CAG triplet that was introduced at the intron-exon junction of intron 5–6, thereby creating a second functional splice acceptor site for exon 6 in frame.

Imaoka *et al*. put forward the intriguing idea that insSer207 in native human CYP4B1 might have occurred to restore the enzyme function by compensating for the negative consequences of the p.P427S alteration that had occurred in humans during evolution [30]. We therefore functionally tested the serine insertion in three different CYP4B1 enzymes with well defined functional activities towards 4-IPO: i) the native rabbit protein, in which serine was artificially introduced at the corresponding amino acid position 202; ii) the native human CYP4B1; and iii) the mutant human CYP4B1 p.S427P enzyme, in which the canonical proline present in other CYP450s at corresponding positions was re-introduced in place of serine. For the latter protein, we recently reported that this p.S427P amino acid change restored the metabolizing activities of the human protein for hallmark substrates of animal CYP4B1 enzymes [28]. The results of the present study clearly demonstrate that insSer207 in the two human proteins and insSer202 in the rabbit enzyme have no functional impact on the bioactivation of 4-IPO in human primary cells and cell lines. In addition, the insertion did not alter the CYP4B1 expression level or half-life when stably expressed in HepG2 cells. Importantly, in contrast to the hypothesis by Imaoka *et al*. [30], insSer207 did not restore the absent enzymatic activity of native human CYP4B1 with serine at position 427 for 4-IPO. Thus, insSer207 in the human mutant p.S427P and insSer202 in the wild-type rabbit CYP4B1 seem to be neutral modifications regarding the xenobiotic metabolizing capabilities of human and animal CYP4B1s.

The homology model for human CYP4B1 mapped insSer207 to a peripheral loop region, and although this localization might support some local structural perturbation, insSer207 clearly does not affect any of the functional read-outs for the three pairs of enzymes studied here (Figs 3–5). In addition, the phylogenetic analysis demonstrated that during evolution, the insertion of a CAG repeat in intron 5 had already occurred at the stage of *hominoidae* and is therefore present in all great apes and humans (data on the *CYP4B1* locus in gibbons as lesser great apes are not available in public databases). As the p.P427S ‘variant’ is unique to humans and arose *later in evolution* than insSer207, the latter has no possible role in rescuing defective human CYP4B1 enzyme activity.

For more than 25 years now, the role and function of the human CYP4B1 has remained a mystery. Retrospectively, this uncertainty seems to be the consequence of a combination of factors, predominantly i) technical difficulties in reliably expressing the native human CYP4B1 enzyme in human cells and thus in performing functional analyses, ii) contradictory results in heterologous expression systems such as insect cells and liver cells of transgenic mice, and iii) the absence of known functions for CYP4B1 in mammals and lower organisms. However, in the last 2 years, we have made considerable progress towards achieving a better understanding of the structure–function relationships for human CYP4B1. Previously, we demonstrated that compared to the very active rabbit CYP4B1 enzyme, the human protein lacks important amino acid determinants in the middle domain of the protein that can render even the human native protein with serine at position 427 able to actively process 4-IPO [28]. Here, we have demonstrated that the two naturally occurring human CYP4B1 isoforms, with/without insSer207, have no 4-IPO bioactivating ability. Therefore, we can safely conclude that the native human CYP4B1 enzyme lost its enzymatic activity against typical xenobiotic pneumotoxins during evolution and therefore is now an ‘orphan’ P450 enzyme in humans. However, it should be noted that it still remains unclear which functional role(s) the active CYP4B1 homologs in great apes and other mammals might have, especially for endogenous substrates.

Finally, the conclusion, that the human CYP4B1 protein, with or without the serine insertion, is unstable, non-functional, and thus redundant in humans fits well with the relatively high frequency of detrimental genetic polymorphisms found in two human studies [44, 45]. Lo-Guidance *et al*. detected five allelic variants of *CYP4B1* in a French Caucasian population with the second most frequent, CYP4B1*2, present in 518 out of 2082 (24.9%) French-Canadian individuals. This allele represents a 2-bp deletion (c.881-2delAT) that results in a prematurely terminated protein [44]. Forty study participants (2%) carried this non-functional allele in the homozygous state [44]. Hiratsuka *et al*. reported that the frequency of a *CYP4B1* allele with the same 2-bp deletion is 33.3% in the Japanese population and that 18 out of 192 (9.4%) individuals are homozygous [45]. These high frequencies of other non-functional *CYP4B1* alleles support the theory that the gene is redundant in humans and therefore not subject to further genetic selection [46].
